# Resonant energy transfer and light scattering enhancement of plasmonic random lasers embedded with silver nanoplates

**DOI:** 10.1039/c9ra10462c

**Published:** 2020-02-19

**Authors:** Jia-Huei Hsiao, Shih-Wen Chen, Bing-Yi Hung, Kasimayan Uma, Wei-Cheng Chen, Chi-Ching Kuo, Ja-Hon Lin

**Affiliations:** National Taipei University of Technology Taipei 106 Taiwan jhlin@ntut.edu.tw; Department of Chemical Engineering and Biotechnology, National Taipei University of Technology Taipei 106 Taiwan; Institute of Organic and Polymeric Materials, National Taipei University of Technology Taipei 106 Taiwan kuocc@mail.ntut.edu.tw

## Abstract

The resonant energy transfer enhancement from a plasmonic random laser (PRL) has been investigated by means of a dye-covered PVA film with embedded silver nanoplates (DC-PVA/AgNPs). Different sizes and morphologies of AgNPs were adopted to shift the localized surface plasmon resonance (LSPR) and intensify recurrent light scattering between the AgNPs. For better overlap between surface plasmon resonance and the photoluminescence of fluorescent molecules with appropriately-sized silver nanoprisms, the slope efficiency of the PRL was greatly enhanced and the lasing threshold was obviously reduced. In addition, the photon lifetime for the DC-PVA/AgNPs film reveals an apparent decline around 1.39 ns owing to better coupling with LSPR. The stronger light scattering of samples with bigger-sized silver nanoprisms has been demonstrated by coherent back scattering measurements, which reveals a smaller transport mean free path around 3.3 μm. With *α*-stable analysis, it has been successfully demonstrated that the tail exponent *α* can be regarded as an identifier of the threshold of random lasing.

## Abstract 2

The resonant energy transfer enhancement from a plasmonic random laser has been investigated by means of a dye-covered PVA film embedded with silver nanoplates with different sizes and morphologies.

## Graphical abstract

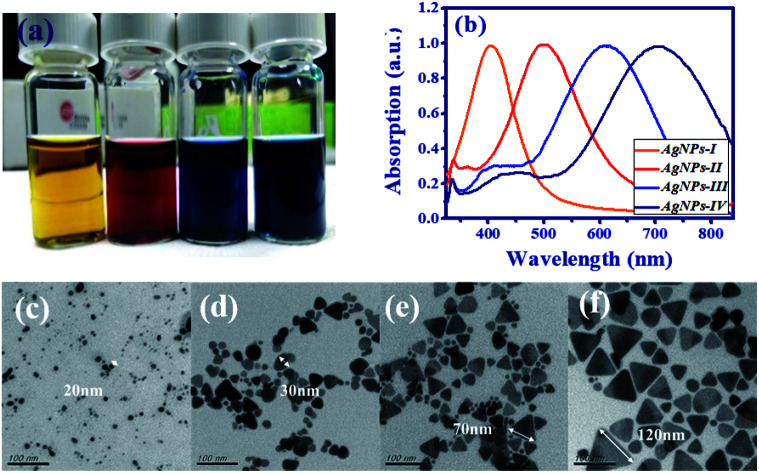

## 1 Introduction

To-date, mirrorless random lasers (RLs),^1,2^ with the characteristics of being compact and flexible with a low lasing threshold, have been widely investigated for several decades. Unlike a traditional laser with a cavity mirror for positive feedback, recurrent light scattering within a disordered alignment of a nanostructure material, plays the dominant role for RLs. Typically, RLs possess unique features like a broad emission angle, multiple discrete emission spikes and low spatial coherence that can be applied in many areas such as single cell imaging,^3^ PH sensing,^4^ biosensors and also in medical diagnosis.^5^ When the condition *l*_s_ ≥ *l*_g_ is reached, RL can be generated.^6^ Here, *l*_s_, scattering mean free path, is defined as the average distance that light travels between two consecutive scattering events, and *l*_g_, amplification length, is inversely proportional to the optical gain. A variety of nanomaterials, such as semiconductor,^7^ dielectric materials,^8^ polymer,^9^ human tissue,^10^ plant structures,^11^ and biological tissues^12^ have been demonstrated to generate RLs. Owing to their intrinsic birefringence, liquid crystals (LCs)^13^ are considered as a superior light scattering material for RLs. In comparison with other scattering materials, the output behavior of RL using dye-doped LCs (DDLCs) can be easily modulated through the temperature, magnetic and electric fields.^14–17^ In addition, DDLCs have been used to fill the inside of capillary fibers or glass cells to reduce their lasing threshold because of the additional optical confinement.^18–20^

Recently, localized surface plasmon resonance (LSPR), a spectacular physical effect, have attracted a great deal of attention to provide strong electromagnetic field confinement or light trapping around the surface of nanostructures. When the incident photon energy matches the coherent electron oscillation frequency,^21^ LSPR will be induced from collective electron oscillation on a surface of noble metallic nanoparticles, such as gold, silver and platinum. LSPR has revealed widespread applications such as photocatalytic activity,^22^ biomedicine for killing tumor tissue, efficiency increase of solar cells,^23,24^ and output intensity enhancement of light emission diodes^25^ and laser diodes.^26,27^ Besides, scientists are also curious about the behavior of plasmonic RLs (PRLs) in combination with laser dyes and metallic NPs within polymer materials. In PRLs, the metallic nanostructure primarily plays the role of an antenna to concentrate the surrounding electric field onto the metallic surface, termed the hot spot, and leads to the enhancement of emission so that lasing efficiency will be greatly improved.^28^ Besides, a certain size and morphology of the metallic nanoparticles will induce more intense plasmon scattering to produce coherent feedback and reduce the lasing threshold. Because of its low chemical degradation and stability property, a number of studies have adopted gold nanoparticles to induce PRL in film^29^ and cellulose nanofibers.^30^ Zhai *et al.*^31^ used the waveguide structure, consisting of the blending of laser dye (DCJTB) and PMMA on top of randomly distributed gold nano-islands, to form the PRL with stronger feedback. In addition, star-shaped gold nanoparticles can localize the field at their spiky tips and reveal better performance for the PRLs than sphere or prolate nanoparticles.^32^ In comparison to gold nanoparticles, silver nanoplates (AgNPs), a kind of two dimensional nanostructure, such as nanoprisms and nanodisks, can produce relatively high electric field confinement and wide range tunability of LSPR in covering almost all the visible spectrum range, depending on their sizes and morphology. Owing to its superior LSPR in the near UV regime in comparison to the gold nanoparticles, a number of studies have focused on the enhancement of RL by means of different morphology AgNPs.^33^ Through mechanical stress, a tunable PRL has been reported from the waveguiding plasmonic gain channel on top of a flexible silicon rubber slab comprising of silver nanowires.^34^

Based on solution preparation strategies using chemical reduction,^35^ the tremendous tailoring ability of the lateral size of AgNPs relative to their thickness can be achieved to shift the wavelength of LSPR. This fact allows us to investigate the PRL characteristics of dye-covered PVA films by embedding different sized AgNPs to alter the LSPR relative to the emission spectrum of the desired active medium. Furthermore, coherent back scattering (CBS) measurements^36^ have been adopted to obtain the transport mean free paths (*l*_t_) of samples by embedding metallic nanoplates to discuss the role of plasmonic feedback or plasmonics scattering on PRL. The time resolved photoluminescence (PL) can help us to discuss the energy coupling between LSPR. Finally, we studied the dynamics of PRL without and with the AgNPs by *α*-stable distribution analysis.^37^

## 2 Preparation of AgNPs and theoretical estimation of LSPR

In this work, AgNPs were synthesized by the solution process,^35^ using the oxidative power and the assistance of an appropriate capping ligand from H_2_O_2_. First, aqueous solution comprising trisodium citrate (Na_3_CA·H_2_O), silver nitrate (AgNO_3_), polyvinylpyrrolidone (PVP), ethanol and hydrogen peroxide (H_2_O_2_), were mixed and stirred in deionized (DI) water at room temperature. Here, PVP and citrate were acted to avoid the accumulation of silver nanoparticles. Then, a suitable reducing agent, *i.e.*, sodium borohydride (NaBH_4_), was added to start a simple reduction reaction. In this preparation process, H_2_O_2_ plays the most indispensable part as an oxidative etchant. It helps to form planar twinned seeds and remove possible non-twinned particles so that silver nanoprisms can be produced eventually.

**Fig. 1:**
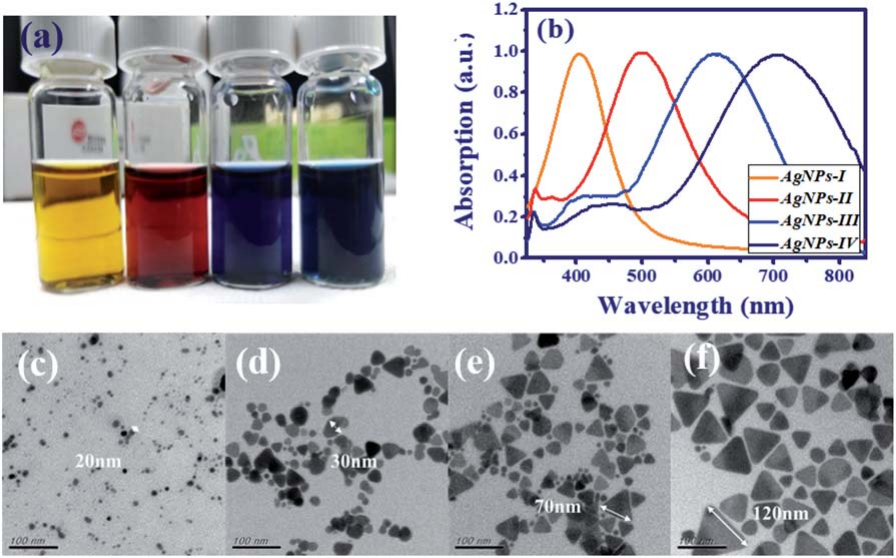
(a) Picture of different sizes of AgNPs suspended in DI water with distinct colors (AgNPs-I: pale yellow, AgNPs-II: red wine, AgNPs-III: indigo, AgNPs-IV: navy), and (b) the corresponding extinction spectra. TEM images of (c) AgNPs-I, (d) AgNPs-II, (e) AgNPs-III, and (f) AgNPs-IV, with different sizes and morphologies of AgNPs.

When the silver nanodisks start to be synthesized, the color of the reaction solution was pale yellow. Following this, we sequentially added a few drops of H_2_O_2_ and sodium borohydride to fine-tune the frequency of the surface plasmon resonance. After a few minutes of stirring, the reaction solution reveals an obvious color change from pale yellow (AgNPs-I) to red wine (AgNPs-II), indigo (AgNPs-III) and navy (AgNPs-IV) as shown in Fig. 1(a). It is an indicator of the production of AgNPs with different sizes and morphologies. The morphologies of the AgNPs were obtained by a transmission electron microscope (TEM, T12 120 keV). For real-time monitoring of the synthesis of silver nanoprisms, the extinction spectrum was obtained by combining a white light source (DH-2000-BAL, Ocean Optics Inc.) and a spectrometer (USB4000 Fiber Optic, Ocean Optic Inc.).

In Fig. 1(b), the extinction spectra indicate that the resonant peak increases from near UV (about 404 nm) to near IR (about 704 nm) as the lateral size of AgNPs increases and the morphology changes. In addition, the TEM images of the four samples (AgNPs-I, AgNPs-II, AgNPs-III, and AgNPs-IV) are shown in Fig. 1(c)–(f). For the smaller size (<20 nm), AgNPs reveal the nanodisk, *e.g.*, AgNPs-I in Fig. 1(c). After adding a few drops of H_2_O_2_ and sodium borohydride, the AgNPs started to aggregate and turn into the nanoprisms in Fig. 1(d)–(f). The size of the nanoprisms increased from 30 nm, *i.e.*, AgNPs-II in Fig. 1(d) to about 120 nm, *i.e.*, AgNPs-IV in Fig. 1(f).

To discuss the enhancement of plasmonic fields, we simulate four different nanoplates using the finite-difference time-domain method (FDTD). FDTD is a numerical technique for solving Maxwell’s differential equations. A set of time-dependent finite difference equations corresponding to eqn (1) and (2) are discretized to space and time partial derivatives by Yee’s cell.^38^1
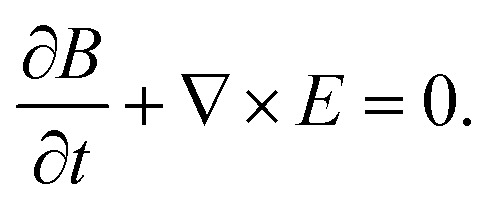
2
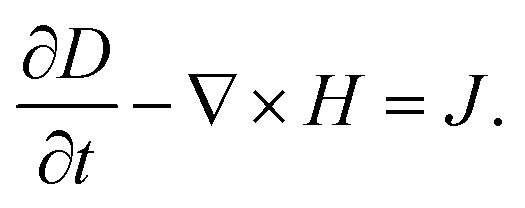


The updated value of the *H*-field in time depends on the value of the last time value of the *H*-field and the curl of the local *E*-field in space. The updated value of the *E*-field in time depends on the value of the last time value of the *E*-field and the curl of the local *H*-field in space. It repeats again and again until the final electromagnetic field is satisfied.

**Fig. 2:**
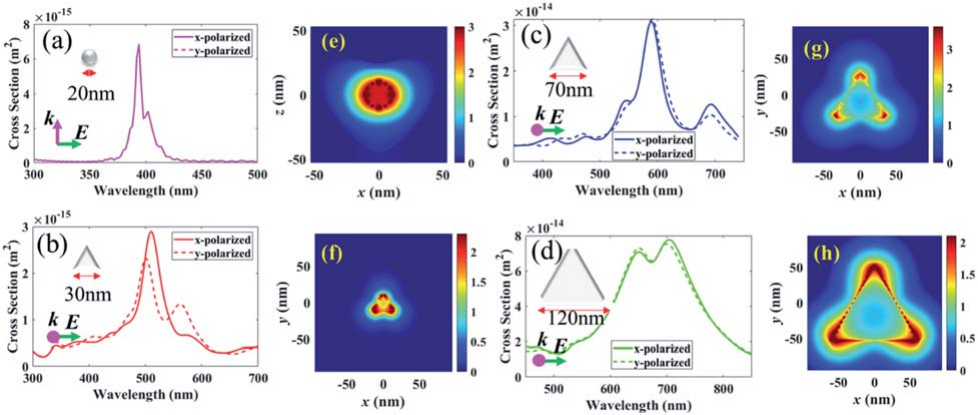
The extinction cross-section spectra of: (a) silver nanosphere with a radius of 10 nm and wavelength of 393 nm; and silver nanoprisms with (b) side edges of 30 nm and wavelengths of 510 nm, (c) side edges of 70 nm and wavelengths of 588 nm, and (d) side edges of 120 nm and wavelengths of 706 nm, respectively (the red arrows indicate the size of Ag). The corresponding enhanced electric field distribution of a nanosphere in (e) and nanoprisms in (f)–(h) (the color bars in (e)–(h) are in log scale).

*x*- and *y*-polarized incident light, was applied to four different nanoplates in the *z*-direction to approach the experimental scenario. One is a silver nanodisk with a radius of 10 nm in Fig. 2(a). The others are silver nanoprisms with side edges of 30 nm, 70 nm, and 120 nm as the red arrows show in Fig. 2(b)–(d), and they have corresponding thicknesses around 20 nm, 40 nm, and 60 nm, respectively. The background refractive index is 1.4, which is an approximation value of the DI water with ion. The simulation domain is 1.4 μm × 1.4 μm × 1.4 μm with boundary conditions of perfectly matched layers^39^ at six facets. After interaction with light, the cross-section of scattering and absorption from a nanoplate is expressed as3
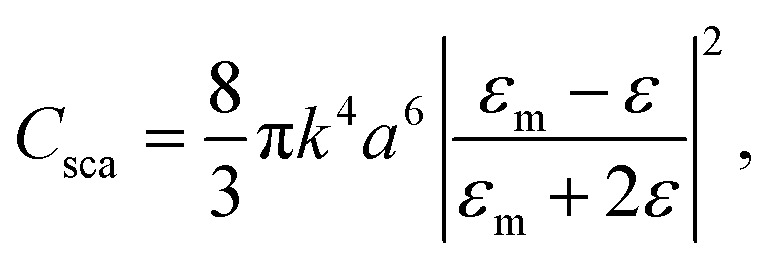
4
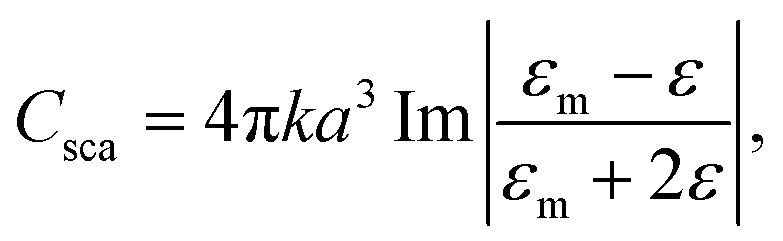
where *a* is the radius of a nanoparticle, *k* is the wavenumber, *ε*_m_ is the permittivity of the metal and *ε* is the permittivity of the background. Extinction of the irradiation includes the absorption and scattering from a nanoplate. A total-field scattered-field technique was adopted to collect the absorption and scattering spectra.

Fig. 2(a)–(d) demonstrate the extinction cross-section spectra, where red arrows indicate the plate sizes. The magenta arrows and green arrows indicate the *k* and *E* directions. Solid and dashed lines indicate the extinction cross section spectra of the *x*-polarized and *y*-polarized incident light, respectively. The resonance peak is affected by the size of the nanoplates and reveals redshift as the size increases. The smallest AgNP is the nanodisk with a resonant peak appearing at 393 nm. For the nanoprism AgNPs with side edges of 30 nm, 70 nm, and 120 nm, the resonant peaks reveal a redshift to 510 nm, 588 nm, and 706 nm. Unpolarized electric field intensities can be obtained from the summation of the *x*-polarized and *y*-polarized electric field intensities. The associated electric field distribution of AgNPs in Fig. 2(a)–(d) (with peak at 393 nm, 510 nm, 588 nm, and 706 nm) are shown in Fig. 2(e)–(h) with the log scale color bar. It is obvious to see the confinement of electric field at the tips of the silver nanoprisms. The intensities reveal more than 100 times enhancement at their resonance frequencies.

## 3 Sample preparation and experimental setup

**Fig. 3:**
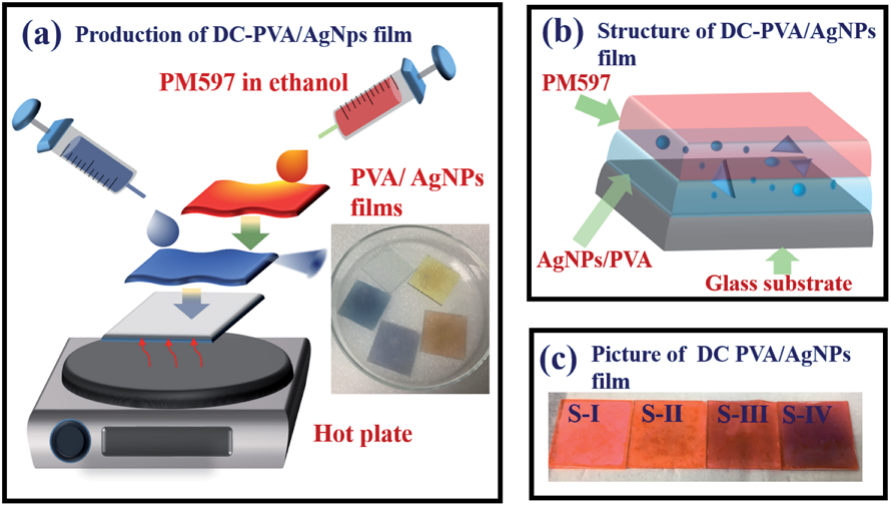
(a) Schematic diagram to describe the preparation process of DC-PVA/AgNPs film (inset: picture of PVA/AgNPs films), (b) structure of DC-PVA/AgNPs film, (c) picture of produced samples S-I, S-II, S-III and S-IV.

A schematic diagram for the preparation process of a dye-covered PVA film with embedded AgNPs (DC-PVA/AgNPs) film is shown in Fig. 3(a). First, the AgNPs solution was homogeneously mixed with PVA solution, which comprises PVA powder and 1 ml distilled water, and then ultra-sonication was performed for about 10 min. Following this, the colloidal solution was spin coated onto a cleaned glass substrate and heated at 40 °C for 40 min. After drying, the laser dye Pyrromethene 597 (PM597, Exciton Inc.) was drop coated on top of the PVA/AgNPs film and dried in an oven at 80 °C for 1 h. The sample structure of the dye covered AgNPs/PVA film is shown in Fig. 3(b) in which the AgNPs were homogeneously embedded in the PVA film. In this work, four samples, *i.e.*, S-I, S-II, S-III, S-IV, were produced that comprise of different sizes of AgNPs, *i.e.*, AgNPs-I, AgNPs-II, AgNP-III, and AgNP-IV, in the PVA film, as shown in Fig. 3(c). It shows that the produced films become darker with larger sized AgNPs because of the redshift of the extinction peak toward a longer wavelength.

**Fig. 4:**
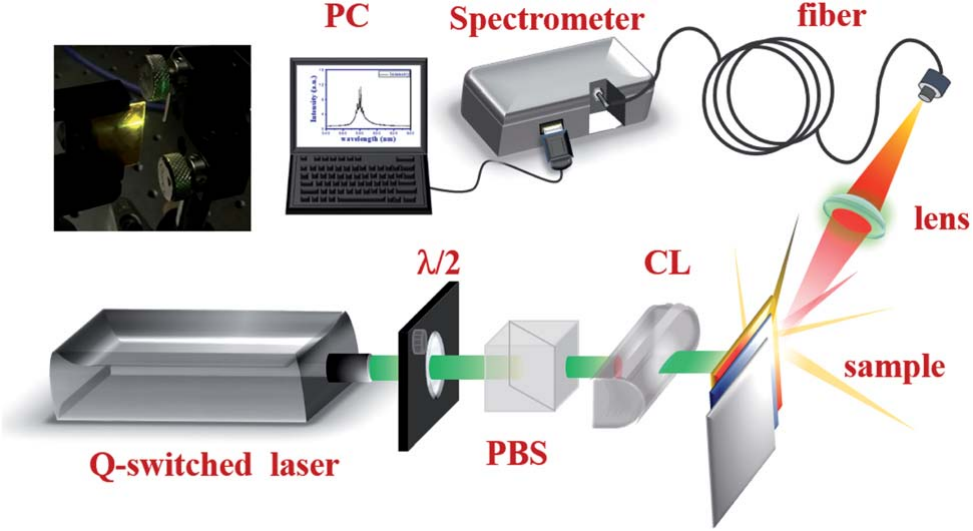
Schematic setup for the generation and measurement of PRL. Here, *λ*/2: half-wave plate, PBS: polarization beam splitter, CL: cylindrical lens, PC: personal computer. Photograph shows the DC-PVA/AgNP film pump with the Q-switched laser.

The experimental setup for the RL generation and measurements from the synthesized DC-PVA/AgNPs films is shown in Fig. 4. A frequency doubling Q-switched Nd:YAG laser (NL200 series, EKSPLA Inc.) with an emission wavelength of 532 nm was used as an excitation source. The pump pulses, having a 10 Hz repetition rate and 2.2 ns pulse duration, were focused onto the sample with a long line stripe (around 6.67 mm × 0.35 mm) by a cylindrical lens with a focal length of 7 cm. The integration of a half-wave plate (*λ*/2) and polarization beam splitter (PBS) controlled the excited pulse energy. The samples were mounted onto a three-dimensional stage. The side emission of the sample was guided by a fiber and measured by an optical spectrometer (HR4000, Ocean Optics Inc.) with a resolution of around 0.3 nm.

The mean free path of the sample was measured by a CBS^36^ through a continuous wave green laser (*λ* = 532 nm) as a light source. The collimated beam was divided into a signal and a reference beam by a BS. The intensity of the signal beam (*I*_sig_), *i.e.*, scattering beamlet from the sample, was recorded by a detector (*D*_sig_). A small pinhole with 0.5 mm diameter was placed in front of the *D*_sig_. The intensity of the reference beam (*I*_ref_) was directly measured by another detector (*D*_R_). In order to obtain the scattering intensity distribution (*I*_sig_/*I*_ref_), the *D*_sig_ was moved in a direction perpendicular to the propagation direction of the incident beam through a translation stage. The PL was measured using a spectrometer (iHR320, Horiba Inc.) equipped with a photomultiplier tube (PMT, R928, Hamamatsu Inc.). A picosecond laser source (LDH-D-C-375, PicoQuant Inc.) with a central wavelength of 372 nm, repetition rate of 10 MHz, and pulse width of 50 ps, was used as an excited source. The photon lifetime was measured by time-correlated single photon counting (TCSPC, TimeHarp 260, PicoQuant Inc.) in combination with a photomultiplier detector assembly (PDA, PicoQuant Inc.) for recording the decay trace.

## 4 Results and discussion

**Fig. 5:**
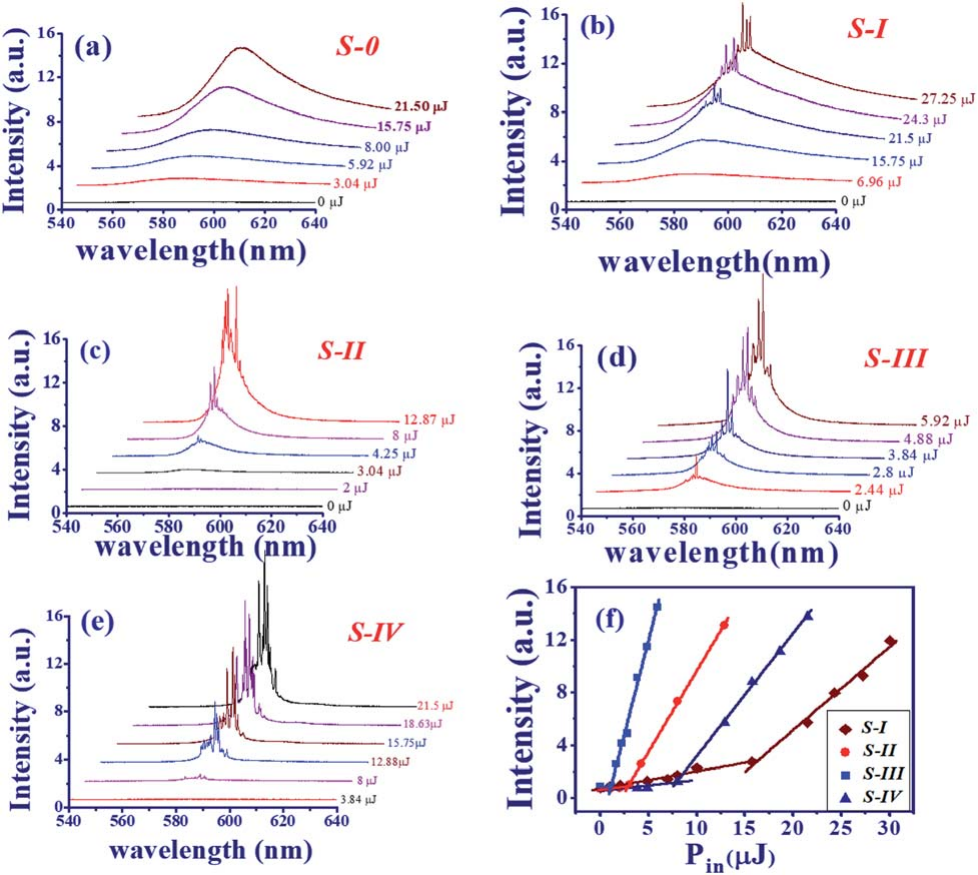
Evolution of emission spectrum *versus* excited energy for the (a) S-0, (b) S-I, (c) S-II, (d) S-III, (e) S-IV, and (f) output intensity *versus* pump energy *P*_in_ (μJ) for four different DC-PVA/AgNP films.

**Fig. 6:**
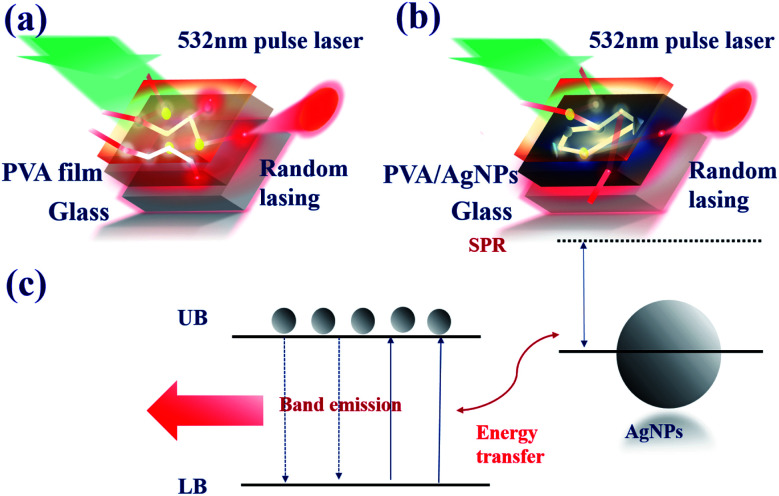
Schematic diagram of light scattering in DC-PVA films (a) without, and (b) with, embedded silver-nanoplates. (c) Energy diagram to illustrate the coupling between the emission of the fluorescent molecule and LSPR of AgNPs.

The evolution of the emission spectra of pure DC-PVA film (S-0) and DC-PVA/AgNPs film (S-I to S-IV) with the increase of excited energy of the Q-switched laser are shown in Fig. 5(a)–(e). If no AgNPs were embedded in the PVA film, the broad emission spectra were revealed at even higher pulse energy (*E*_p_ = 21.5 μJ) in Fig. 5(a). Owing to the incoherent feedback of light scattering between the laser dye and PVA film as shown in Fig. 6(a), the emission bandwidth of S-0 decreased slightly from 57.7 nm to 39.4 nm as pump energy *E*_p_ increased from 3.9 μJ to 21.5 μJ. As we embed silver nanodisks into the PVA film, a number of aperiodic emission spikes were excited on top of the broad emission spectrum as the pump energy increased above 21.5 μJ, Fig. 5(b). It is attributed to recurrent light scattering (or plasmonic light scattering) between the AgNPs to form a number of closed-loops, termed the coherent feedback, which can be schematically described in Fig. 6(b). If we further increased the pump energy, more spatial modes were excited because more photons gain enough optical amplification from fluorescent molecules in their scattering light track to reach the lasing threshold. Thus, the number and the intensity of the emission spike increased at higher pump energy.

In comparison to Fig. 5(b), the emission spike of S-II and S-III, with larger size nanoprisms embedded, emerged at lower excited energy as shown in Fig. 5(c) and (d). Especially, the output property such as the intensity of emission spikes and pedestal in Fig. 5(d) revealed an impressive improvement in comparison to other samples. Owing to the better overlap of the emission spectrum of gain medium and the extinction peak of the AgNPs-III (blue curve in Fig. 1(f)), the LSPR effect will enhance the energy coupling between plasmonic resonance and electron hole recombination of fluorescent molecules. In addition, the width of the pedestal from S-III is slightly narrower than that from S-II which can also result from the enhancement of the plasmonic scattering between the disordered alignment of the larger size nanoprism AgNPs. For this reason, S-IV shows the greatest number of emission spikes in Fig. 5(e) because of the largest size of nanoprism out of all the samples.

Fig. 5(f) illustrates the intensity of the maximum emission spikes as a function of the pump energy for all the samples (S-I: brown prisms, S-II: red circles, S-III: blue squares, and S-IV: navy triangles). As the pulse energy increases, two slopes can be obtained to illustrate the spontaneous and stimulated emission, respectively. For the S-I, with the smallest size AgNPs, it reveals the lowest slow efficiency and the highest lasing threshold of about 15.75 μJ, corresponding to an energy density about 0.675 mJ cm^−2^. As the size of AgNPs increased or more nanoprism structures were embedded, the RL revealed higher slope efficiency and a lower lasing threshold. S-III shows the highest slope efficiency and lowest lasing threshold of about 1.12 μJ, corresponding to an energy density of about 0.048 mJ cm^−2^. Nevertheless, the threshold increases and slope efficiency declines for S-IV.

The schematic diagram of the band emission of fluorescent molecules and LSPR of AgNPs is shown in Fig. 6(c). While the frequency of emission light from the dye molecules is near the plasmon resonance at the surface of the AgNPs, the intensity of the electron oscillations increases obviously. The electric field will be confined at the tip of the silver nanoprism as shown in Fig. 2. Then, more energy will transfer back or couple to the fluorescent dye molecule to excite more electrons from the lower band (LB) to the upper band (UB). In this work, the energy coupling between AgNP-III (blue solid curve, Fig. 1(f)) to the light emission of fluorescent dye molecules (inset of Fig. 8) is better than the others. Thus it will induce more efficient electron–hole recombinations for the increase in emission intensity. Owing to the decrease in the overlap between SPR and the lasing emission peak, the lasing threshold increases and the slope efficiency declines for S-IV compared with S-III.

**Fig. 7:**
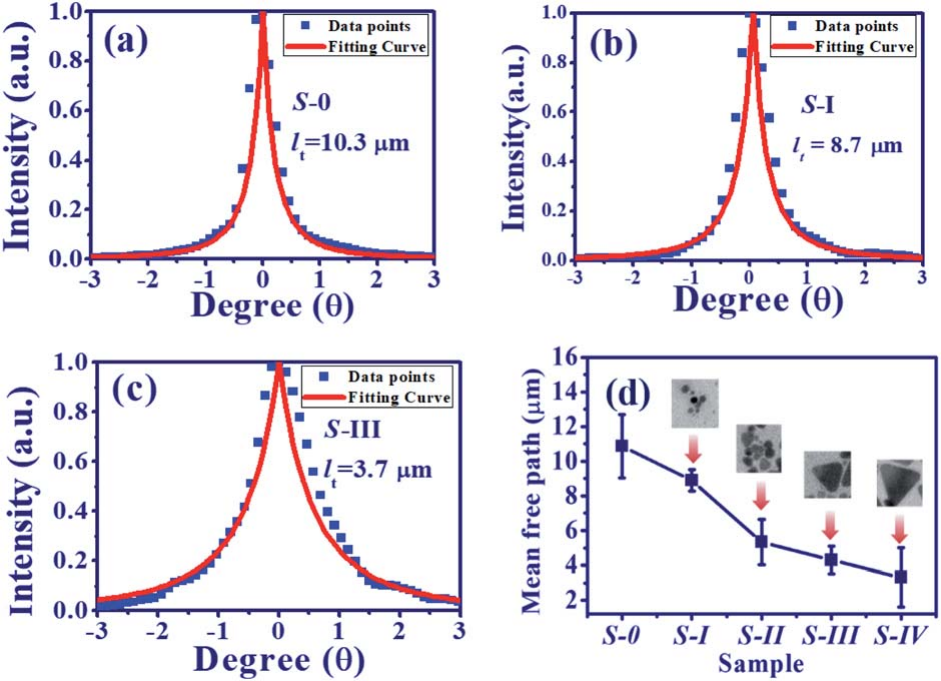
CBS cone for (a) S-0, (b) S-I, and (c) S-III, (blue squares: measured data, red solid line: theoretical fitting curve). (d) Transport mean free path from all the samples (inset figures: TEM images of the AgNPs).

In addition to the SPR, the plasmonic scattering between disordering alignment of AgNPs also plays a dominant role in the output characteristic of PRL. Typically, the event of light scattering in random media can be classified into three regimes, *i.e.*, localized (*l*_s_ ≤ *λ*), diffusive (*λ* ≤ *l*_s_ ≤ *L*), and the weak (*L* ≤ *l*_s_) scattering regime, where *λ* is the wavelength and *L* is the sample size.^6^ Similar to the Anderson localization for the electron confinement,^40^ intensely recurrent light scattering causes highly spatial confinement of photons. As shown in Fig. 6(b), the scattering light trajectory can form a closed loop to produce coherent feedback. Under this condition, the interference leads to standing-wave patterns and creates specific modes. It is the reason that multiple coherent spikes emerged on top of the pedestal in Fig. 5(b)–(e). In order to quantitatively characterize the light scattering in the medium, the transport mean free path *l*_t_ was measured using CBS.^36^ Here, *l*_t_ is defined as the average travelling distance of a light wave before propagation direction becomes randomized. Fig. 7(a)–(c) illustrate the scattering light intensity distribution (blue squares) of S-0, S-I, and S-III as a function of the divergence angle from the CBS measurements. The CBS cone of S-0, DC-PVA film without AgNPs, in Fig. 7(a) shows the narrowest diverged angle and the largest means free path. After embedding AgNPs inside the PVA film, the CBS cone increased obviously, *e.g.*, S-I and S-III in Fig. 7(b) and (c), respectively, in comparison to the S-0. It indicates that the intensity of light scattering can be efficiently raised through plasmonics scattering within the DC-PVA film with AgNPs.

Theoretically, the CBS cone can be estimated using the following equation:^41^5
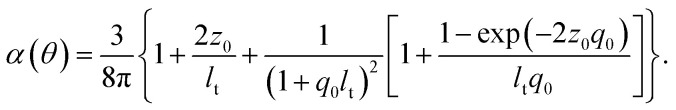
Here, *q*_0_ = 2π*θ*/*λ* is the component of *q* normal to the *z*-axis, and *z*_0_ ≅ 0.7*l*_t_ (for pointlike scatter). Using eqn (5), the CBS cones were well fitted as shown by the red solid curves in Fig. 7(a)–(c). The transport mean free paths of the samples are illustrated in Fig. 7(d) with the corresponding TEM image of the AgNPs shown in the insets. It is obvious to see that *l*_t_ declines from 10.3 μm for the S-0, without AgNPs, to the smallest value of about 3.3 μm for S-IV, with the embedding of the largest size of silver nanoprism (AgNPs-IV). This result accounts for the fact that the size and morphology of the AgNPs can efficiently change light scattering.

**Fig. 8:**
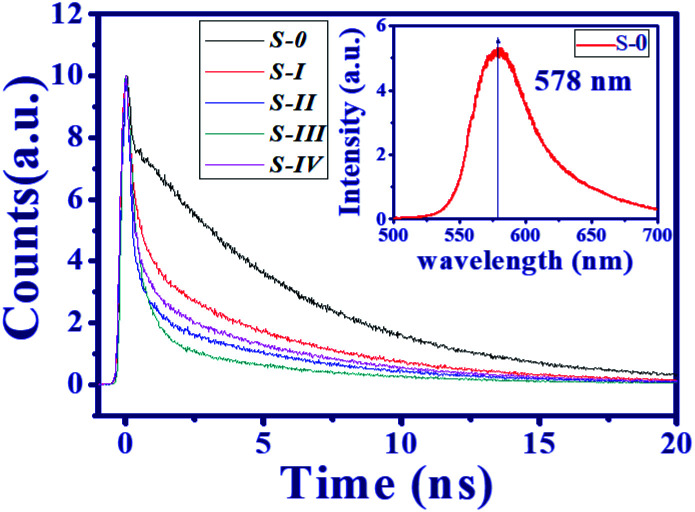
Decay trace of DC-PVA films without (S-0: black), and with AgNPs (S-I: red line, S-II: blue line, S-III: green line, and S-IV: pink line) through TCSPC measurements (inset figure: PL spectrum of the pure DC-PVA film).

To further understand the mechanism of illumination enhancement of the DC-PVA film after embedding it with AgNPs, a time-resolved PL measurement was performed to obtain the photon lifetime from the radiation recombination of the excited dye molecules. In this work, the integrated PL spectrum of all the produced samples are similar, *e.g.*, the spectrum of S-0 with a pump power of 0.3 mW (inset of Fig. 8(a)), reveals the maximum emission peak at 578 nm. Fig. 8 illustrates the decay profile of all the produced samples at 578 nm (S-0: black line, S-I: red line, S-II: blue line, S-III: green line, S-IV: pink line). For S-0, a relatively fast decay trace below 0.35 ns delay time is contributed to by the residual UV pump pulse. In addition, a slow decay trace can be fitted by the single exponential function to obtain the decay time of approximately 6.02 ns. It accounts for the radiation recombination of the fluorescent molecules.

In comparison to S-0, the decay traces of the DC-PVA films with embedded AgNPs (S-I to S-IV) in Fig. 8 become faster and can be fitted by the bi-exponential model:6
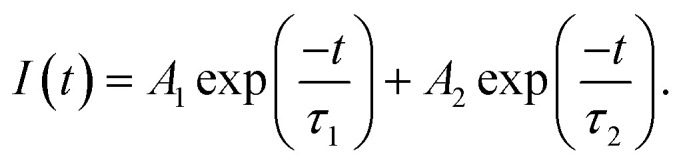
Here, *τ*_1_ and *τ*_2_ are the fast and slow decay time constant with the corresponding weighting factors *A*_1_ and *A*_2_. Table 1 lists all the fitting parameters of the samples. It is noted that the *τ*_1_ and *τ*_2_ of the DC-PVA/AgNP films are almost the same but the weight factors vary considerably. The values of *τ*_2_ (slow) from the DC-PVA/AgNPs films are close to S-0 (no AgNPs), which mainly results from the radiative recombination of PM597. Besides, the shorter lifetime *τ*_1_ (fast) around 0.5 ns is contributed to by the coupling between the photon emission of dye molecules and the plasmon resonance. The average lifetime (*τ*_avg_) was calculated according to:^25^

7 *τ*_ave_ = (*A*_1_*τ*_1_ + *A*_2_*τ*_2_)/(*A*_1_ + *A*_2_).

**Table 1:** Fitting parameters of the DC-PVA film by eqn (6)

Sample	*A*_1_	*τ*_1_ (ns)	*A*_2_	*τ*_2_ (ns)	*A*_1_/*A*_2_	*τ*_ave_ (ns)
S-0			4119.4	6.02		6.02
S-I	3880.6	0.49	4107.3	5.68	0.94	3.16
S-II	3336.5	0.51	2450.4	5.64	1.36	2.68
S-III	6904.5	0.50	1594.7	5.25	4.33	1.39
S-IV	3680.3	0.51	3057.0	5.71	1.2	2.87

Owing to the increased ratio of *A*_1_/*A*_2_, the S-III reveals the shortest average lifetime of about 1.39 ns as shown in Table 1. As the overlap between the spontaneous emission of fluorescent molecules and the electron oscillation frequency on the metallic surface increases, the local electric field confinement in the tip of the nanoprism will be enhanced as shown in Fig. 2(g). The energy coupling between the plasmon resonance and emission of fluorescent molecules might shrink the electron recombination time from the excited state to the ground state and accelerate the radiative decay.

**Fig. 9:**
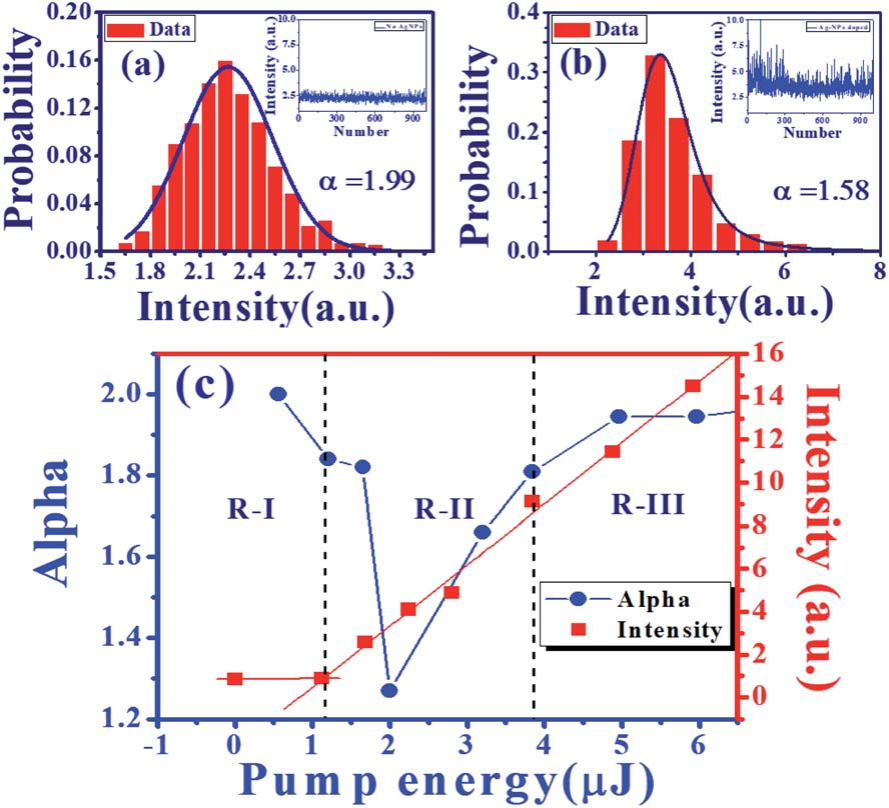
Probability distribution of intensity fluctuation at 578 nm (red histograms) and the theoretical fitting curves (navy solid curves) from (a) DC-PVA film with *α* = 1.99, and (b) DC-PVA/AgNPs film (S-III) with *α* = 1.58. (c) The value of exponent *α* (blue circles) and output intensity (red squares) of S-III as a function of pump energies.

In a previous work, Uppu *et al.*^37^ demonstrated that the tail exponent (*α*) of the stable distribution is a clear indicator of the threshold of random lasing. In order to analyze the characteristics of a RL, a total of 1000 spectra points from samples were captured for the *α*-stable distribution analysis.^42^ The inset of Fig. 9(a) and (b) show the intensity fluctuation of RL from S-0 (pure DC-PVA film) and S-III (DC-PVA/AgNPs film), respectively, with an emission wavelength at 578 nm. The probability of intensity distribution is shown in the red histogram. Using the theoretical fitting of the *α*-stable distribution function in the supporting data, the tail exponent *α* from S-0 and S-III are about 1.99 and 1.58, respectively, which indicates the Gaussian (*α* ≈ 2) and Lévy behavior (*α* < 2), respectively. After embedding AgNPs, more intense light scattering was induced to result in a number of closed loops and the amplification of coherent modes. Thus, more strong intensity fluctuation causes Gaussian distribution to be out of balance and extends the tail exponent of distribution.

Furthermore, we obtained the tail exponent *α* of S-III at different levels of excited energy. The evolution of *α* (blue circles) and peak intensity (red squares) as a function of excited energy is shown in Fig. 9. Like previous reports, as excited energy increases, three distinct regimes (R-I, R-II, R-III) can be discovered from the Gaussian behavior followed by Lévy statistics, and then recovered to the Gaussian statistics again. At lower pump pulse energy (R-I), the gain is lower than the loss, the coherent modes or the emission spike cannot be excited, which represents a near Gaussian behavior (*α* > 1.8). When the RL crosses the threshold (*E*_p_ = 1.12 μJ, R-II), the RL starts to be generated and shows large intensity fluctuations. Then, the *α* declines abruptly and shows the smallest value around 1.27 with a heavy tail. When the excitation energy increases (*E*_p_ > 2 μJ), the *α* starts to increase with more excited coherent mode generation. Owing to the average of huge coherent modes, the Lévy distribution returns to Gauss distribution again in R-III (*E*_p_ > 3.84 μJ).

## 5 Conclusions

In this work, the dynamics of the PRL made from a DC-PVA film with different sizes and morphology of AgNPs embedded, has been investigated. Here, the AgNPs were synthesized by a redox method to reveal that the nanodisk for the smaller size turns into a nanoprism when the size increases above 20 nm. As the size of the nanoplates increases, the LSPR of AgNPs shifts from near UV to near IR. Through the excitation of the Q-switched laser, several emission spikes were excited on the top of the pedestal for the DC-PVA film embedded with AgNPs. In addition, the number of spikes increases with the size of the nanoprism because there is more intense light scattering for the larger size silver nanoprism, which reveals a smaller transport mean free path using measurements of CBS. For the excellent overlap between the plasmon resonance of AgNPs and the PL of fluorescent molecules, the slope efficiency of PRL was enhanced obviously and the lasing threshold reduced greatly. In addition, the photon lifetime of the sample with the highest LSPR effect reveals a fast decay trace that can be attributed to the energy coupling between electron oscillation to the interband transition of fluorescent molecules to speed up the recombination process. Finally, the *α*-stable distribution was used to quantitatively determine the characteristics of PRL from DC-PVA film with and without AgNPs to reveal the Gaussian and Lévy distribution, respectively. The tail exponent *α* can also be regarded as an identifier of the threshold of random lasing to reveal three regimes, *i.e.*, Gaussian, Lévy and Gaussian behavior, as the pump excited energy increases.

## Conflicts of interest

There are no conflicts to declare.

## Supplementary Material
